# Genetic diversity of hepatitis B virus quasispecies in different biological compartments reveals distinct genotypes

**DOI:** 10.1038/s41598-023-43655-0

**Published:** 2023-10-09

**Authors:** Bárbara Vieira do Lago, Cristianne Sousa Bezerra, Daniel Andrade Moreira, Thiago Estevam Parente, Moyra Machado Portilho, Rodrigo Pessôa, Sabri Saeed Sanabani, Livia Melo Villar

**Affiliations:** 1 Laboratório de Hepatites Virais, Instituto Oswaldo Cruz, Fundação Oswaldo Cruz, Rio de Janeiro, Rio de Janeiro Brazil; 2 Departamento de Educação, Instituto Federal de Educação, Ciência e Tecnologia do Ceará, Fortaleza, Ceará Brazil; 3 Laboratório de Genômica Aplicada e Bioinovações, Instituto Oswaldo Cruz, Fundação Oswaldo Cruz, Rio de Janeiro, Brazil; 4 Instituto Gonçalo Moniz, Fundação Oswaldo Cruz, Salvador, BA Brazil; 5 Postgraduate Program in Translational Medicine, Department of Medicine, Federal University of Sao Paulo (UNIFESP), São Paulo, Brazil; 6 Laboratory of Medical Investigation (LIM) 03, Clinics Hospital, Faculty of Medicine, University of São Paulo, São Paulo, Brazil

**Keywords:** Computational biology and bioinformatics, Evolution, Microbiology, Molecular biology, Diseases, Risk factors

## Abstract

The selection pressure imposed by the host immune system impacts hepatitis B virus (HBV) quasispecies variability. This study evaluates HBV genetic diversity in different biological fluids. Twenty paired serum, oral fluid, and DBS samples from chronic HBV carriers were analyzed using both Sanger and next generation sequencing (NGS). The mean HBV viral load in serum was 5.19 ± 4.3 log IU/mL (median 5.29, IQR 3.01–7.93). Genotype distribution was: HBV/A1 55% (11/20), A2 15% (3/20), D3 10% (2/20), F2 15% (3/20), and F4 5% (1/20). Genotype agreement between serum and oral fluid was 100% (genetic distances 0.0–0.006), while that between serum and DBS was 80% (genetic distances 0.0–0.115). Two individuals presented discordant genotypes in serum and DBS. Minor population analysis revealed a mixed population. All samples displayed mutations in polymerase and/or surface genes. Major population analysis of the polymerase pointed to positions H122 and M129 as the most polymorphic (≥ 75% variability), followed by V163 (55%) and I253 (50%). Neither Sanger nor NGS detected any antiviral primary resistance mutations in the major populations. Minor population analysis, however, demonstrated the rtM204I resistance mutation in all individuals, ranging from 2.8 to 7.5% in serum, 2.5 to 6.3% in oral fluid, and 3.6 to 7.2% in DBS. This study demonstrated that different fluids can be used to assess HBV diversity, nonetheless, genotypic differences according to biological compartments can be observed.

## Introduction

Hepatitis B virus (HBV), the prototype member of the *Hepadnaviridae* family, is a partially double stranded DNA virus with a complex replication strategy. HBV biosynthesis employs an error-prone polymerase responsible for several replicative steps, including the reverse transcription of an intermediate RNA^1^. HBV is notable for the enormous accumulation of genetic variation during the course of an infection. This variation can be explained by a number of factors, including the organization of the viral genome, the high rate of viral turnover (more than 10^11^ virions each day), the infectivity of the virus, and the recombination events that occur when the virus is replicating. As a result, the HBV population is composed of a swarm of variants that are closely linked genetically; these variants constitute a quasispecies that is able to swiftly adapt to a variety of selective pressures^1–3^. The striking viral genetic variability has led to10 phylogenetic distantly related genotypes (A to J) and more than 35 subgenotypes^2^. As a result, the HBV population consists of closely related but not identical isolates called quasispecies. The composition of the viral quasispecies in a host evolves over time and may fluctuate according to the body reservoir due to specific selective pressure promoted by the host immune response^3^. The genotypic and genetic variability of viral quasispecies has been implicated in distinct prognoses during chronic infection. Specific HBV variants may be linked to an increased risk of severe liver disease, such as cirrhosis and hepatocarcinoma (HCC)^4,5^.

Regarding HBV genetic variability analyses, direct Sanger sequencing is useful for identifying the predominant strains circulating in a sample but cannot detect the heterogeneous profile of viral quasispecies. Next-generation sequencing (NGS) is a high throughput method that has been used to characterize the genomes of genetically diverseviral isolates, including minor viral populations in a comprehensive way that is not possible with traditional methodologies such as Sanger sequencing or cloning.

Previous studies have demonstrated the applicability of dried blood spot (DBS) and oral fluid samples as convenient alternatives for HBV detection and sequencing^6,7^. They are less invasive, easier to collect, require minimal training and may be useful for accessing HBV diversity in settings where blood collection is difficult.

The aim of this study was to evaluate the genetic diversity of HBV quasispecies in various body fluids from chronically infected patients living in Brazil, a country with significant ethnic admixture that is directly reflected in the genetic variability of circulating HBV isolates.

## Materials and methods

### Studied population

Between July 2013 and September 2015, patients were recruited at public health centers in Southeast, South, and Northeast Brazil. Twenty chronic HBV infected patients, who had complex viral molecular profiles (more than 2 nonsynonymous mutations) from previous studies^6,7^ provided serum samples to this study. Twelve of these patients also provided paired samples of oral fluid (saliva) and sixteen provided paired dried blood spot (DBS) samples. As the specimens employed in this study come from previous studies, the methodology regarding the recruitment of the participants is described elsewhere^6,7^.

All 20 individuals had detectable HBsAg and HBV DNA in their serum, 10 had detectable anti-HBe, and 9 had detectable HBeAg in serum (missing information for one of them). Six of the 20 patients were receiving HBV treatment, while the remaining 14 were not. Informed consent was obtained from all participants prior to sample collection. This study was approved by the Ethics Committee of the Oswaldo Cruz Foundation (number 661.187/CAAE 18281313.4.0000.5248). All procedures were performed in accordance with the ethical standards of the responsible committee on human experimentation (institutional and national) and with the Helsinki Declaration of 1975, revised in 2008.

### Sample collection

Blood samples were collected by venipuncture and paired serum and DBS samples were obtained. To prepare DBS samples, seventy-five microliters of whole blood was spotted on Whatman 903 paper (GE Healthcare, Life Sciences, Marlborough, MA)^8^. Each DBS sample was air-dried at room temperature for 4 h and stored in a zip-locked bag with a silica gel desiccant sachet at − 20 °C.

Oral fluid samples were obtained using a Salivette collector (Sarstedt, Nümbrecht, Germany) as previously described^9,10^. Participants were instructed not to eat or drink for 60 min before the exam and to rinse their mouths with water prior to oral fluid collection to remove debris. Additionally, oral fluid samples were visually checked for blood contamination and excluded if observed.

### HBV DNA extraction and quantification

HBV DNA was extracted from serum samples using the High Pure Viral Nucleic Acid Kit (Roche Diagnostics, Mannheim, Germany) following the manufacturer's instructions. The same kit was used for HBV DNA extraction from DBS, according to Bezerra et al.^8^. Three circles of 3 mm of DBS were used directly in DNA extraction and all the steps recommended by the manufacturer were followed. The DNA was eluted in 50 µL of elution buffer. HBV DNA extraction from oral fluid was performed using the “RTP^®^ DNA/RNA Virus Mini Kit” (Stratec, Birkenfeld, Germany) following the manufacturer’s instructions with an increased sample volume (400 µL) of oral fluid^11^.

HBV-DNA was measured in serum samples using the Abbott Real Time HBV (Abbott Diagnostics, Des Moines, USA) assay, followingthe manufacturer’s instructions.

### HBV amplification and sequence analysis

For HBV amplification (900 base pairs), optimized PCR was performed using oligonucleotides for the overlapping S/polymerase genes (genomic position of primers: HBV_1F: 180–203; HBV_4R: 1120–1100)^12^ in accordance with the procedure described by Portilho et al*.*^10^.

After purification with the QIAquick gel extraction kit (Qiagen, Hilden, Germany), the PCR products were used for sequence analysis. Direct nucleotide sequencing reactions were performed in both directions using a Big Dye Terminator kit (version 3.1, Applied Biosystems, Foster City, CA, USA) with external and internal oligonucleotides^12^. Sequencing reactions were run on an ABI3730 automated sequencer (Applied Biosystems, USA)aligned and analyzed by using Clustal W program implemented in MEGA software version 7.0^13^.

### Library preparation for high-throughput sequencing

The sequencing libraries were prepared as previously described^14^. Briefly, one nanogram of each sample amplicon was used in a fragmentation reaction mix employing the Nextera XT DNA Sample Prep kit (Illumina, San Diego, CA, USA) according to the manufacturer's protocol. Tagmentation and fragmentation of each sample were simultaneously performed by incubation for 5 min at 55 °C followed by incubation in a neutralizing tagment buffer for 5 min at room temperature. After neutralization of the fragmented DNA, light 12-cycle PCR was performed with the Illumina Ready Mix to add Illumina flowcell adaptors, indexes, and common adapters for cluster generation and sequencing. An amplified DNA library was purified subsequently using Agencourt AMPure XP beads (Beckman Coulter, Inc., USA), which excluded very short library fragments. Prior to cluster generation, normalized libraries were quantified by real-time PCR (qPCR) using the SYBR fast Illumina library quantification kit (KAPA Biosystems, Woburn, MA, USA) following the instructions of the manufacturer. qPCR was run on the 7500 Fast Real-Time PCR System (Applied Biosystems, USA). The thermocycling and denaturation conditions were detailed previously^15^. Finally, the prepared libraries were loaded on an Illumina MiSeq clamshell cartridge for paired-end 250 sequencing.

### Sequence analyses of NGS

Assembly and data analysis were performed as previously described^15^. Briefly, fastq files were generated by the Illumina MiSeq reporter and validated to assess the distribution of quality scores (Illumina BaseSpace). Due to the sequencing error rate, we only considered variants detected at a frequency higher than 1% and Phred quality score of > 30 (base call accuracy of 99.9%). Validated fastq files from each viral genome were de novo assembled into contiguous sequences and annotated with CLC Genomics Workbench version 5.5 (QIAgen) with default parameters.

HBV quasispecies heterogeneity was assessed based on viral genetic complexity and diversity. Parameters evaluated in diversity analysis were (i) HBV mean genetic distance, (ii) the number of synonymous substitutions per synonymous site (dS), and (iii) the number of nonsynonymous substitutions per nonsynonymous site (dN). Shannon entropy (a measure of uncertainty/variability) and mutation frequency were employed to calculate the complexity of nucleotides and amino acids in S/Pol genes using MEGA software version 7.0^13^.

Bioinformatic analyzes were performed on the Fiocruz Bioinformatic Platform Highz (Fiocruz Rio—RPT04) server. Read quality was evaluated with FastQC v0.11.9 (Babraham Bioinformatics Institute)^16^ and MultiQc v1.7^17^. Reads were then trimmed and quality filtered using Trimmomatic v0.39^18^.

Bwa mem v0.7.17^19^ was used to map reads against HBV virus genome (GenBank: X02763.1) and reformatted using SAMtools v1.10^20^. The consensus sequence was subsequently generated from contigs of each sample using using iVar v1.3.1^21^. To identify nucleotide variants and different haplotypes in a single sample, the reads of each sample were mapped against its own consensus sequence. Duplicate read mappings were marked and then removed using sambamba v0.8.0^22^. Subsequently, iVar v1.3.1, mentioned above, was then used to call nucleotide variants. Haplotype analysis for each sample were performed with Cliquesnv v2.0.3^23^. Only haplotypes with frequency higher than 1% were kept for further analysis. Shannon entropy (H =  − ∑(pilog(pi))) was calculated for each sample and for each nucleotide/amino acid residue, for which pi were either the frequency of each haplotype among the viral quasispecies population or the frequency of each variant called. Nucleotide diversity was calculated by the average pairwise genetic distance between the identified haplotypes (MEGA v11.0.11). Figures were generated with ggplot2 R package^24^.

### Phylogenetic analysis of Sanger sequences and NGS major populations

The evolutionary relationships between the newly generated consensus sequences and 68 sequences representative of all HBV genotypes/subgenotypes retrieved from GenBank (S/POL genes) were determined by phylogenetic tree analysis. Accession numbers of the reference sequences are displayed in the phylogenetic tree and in the supplementary file. Phylogenies were reconstructed through the maximum likelihood (ML) method using the GTR + I + G nucleotide substitution model which was selected as the best-fit model according to the Akaike information criterion in jModelTest 2 program (PMID: 22847109). Genetic distances between and within sequences were evaluated based on nucleotide ‘p’ distances in the MEGA 7.0 program^13^ with bootstrap resampling of 1000 replicates.

### Ethics approval and consent to participate

The study was approved by the Ethical Committee of the Oswaldo Cruz Foundation (number 661.187/CAAE 18281313.4.0000.5248). All procedures were performed in accordance with the ethical standards of the responsible committee on human experimentation (institutional and national) and with the Helsinki Declaration.

## Results

### Study population

Twenty HBV chronically infected individuals provided serum samples for HBV sequence analysis. Twelve of these patients also provided oral fluid, while sixteen also provided DBS samples. Seven participants provided all three biological samples (serum, DBS and oral fluid). Regarding the demographic characteristics, 12 participants were female (12/20; 60%) and the mean age (± SD) was 45.5 ± 27.5 years (median age was 46.5, interquartile range [IQR] 36.25–53.25; 17). Six participants were under antiviral therapy. The demographic, laboratory and clinical characteristics of the individuals are shown in Table 1.

**Table 1:** Demographic, biochemical and serological profiles of the study individuals.

Patient	Gender	Age range	ALT		AST	Therapy	Sample	HBe/anti-HBe	HBs/anti-HBs	Anti-HBc total/IgM	Coinfections
TSO 1808	M	30–39	10	7		No	Serum	−/+	+/−	+/−	No
TSO 1810	F	40–49	11	5		No	Serum	−/+	+/−	+/−	No
TSO 1823	F	60–69	ND	ND		ND	Serum	−/+	+/−	+/−	No
TSO 1828	M	40–49	12	11		No	Serum, DBS, OF	+/−	+/−	+/−	HIV +
TSO 1837	F	50–59	42	19		No	Serum, DBS, OF	−/+	+/−	+/−	No
TSO 1855	M	50–59	20	17		No	Serum, DBS, OF	−/+	+/−	+/+	No
TSO 1941	F	30–39	9	19		Yes	Serum, OF	+/−	+/−	+/−	No
TSO 1948	M	20–29	ND	ND		ND	Serum, OF	+/−	+/−	+/−	No
TSO 2055	F	30–39	13	11		No	Serum	−/+	+/−	+/−	No
TSO 2056	F	20–29	26	42		No	Serum, DBS, OF	+/−	+/−	+/−	No
TSO 2063	F	50–59	7	12		Yes	Serum	−/+	+/−	+/−	No
TSO 2064	F	20–29	27	26		No	Serum, DBS	−/+	+/−	+/−	No
TSO 2065	F	60–69	12	14		No	Serum, DBS	−/+	+/−	+/−	No
TSO 2069	M	50–59	7	21		No	Serum	+/−	+/−	+/−	No
TSO 2072	M	40–49	14	52		Yes	Serum, DBS	+/−	+/−	+/−	No
TSO 2073	M	40–49	12	34		Yes	Serum	−/+	+/−	+/−	No
TSO 2201	M	40–49	ND	ND		Yes	Serum, OF	ND	+/−	+/−	No
LC 04	M	20–29	9	13		Yes	Serum, DBS	+/−	+/−	+/−	No
LC 43	F	70–79	21	44		No	Serum, DBS	−/+	+	+/−	No
LC 87	F	60–69	15	22		Yes	Serum	+/−	+	+/+	No

*ND* No data.

### Molecular tests and sequencing

**Figure 1:**
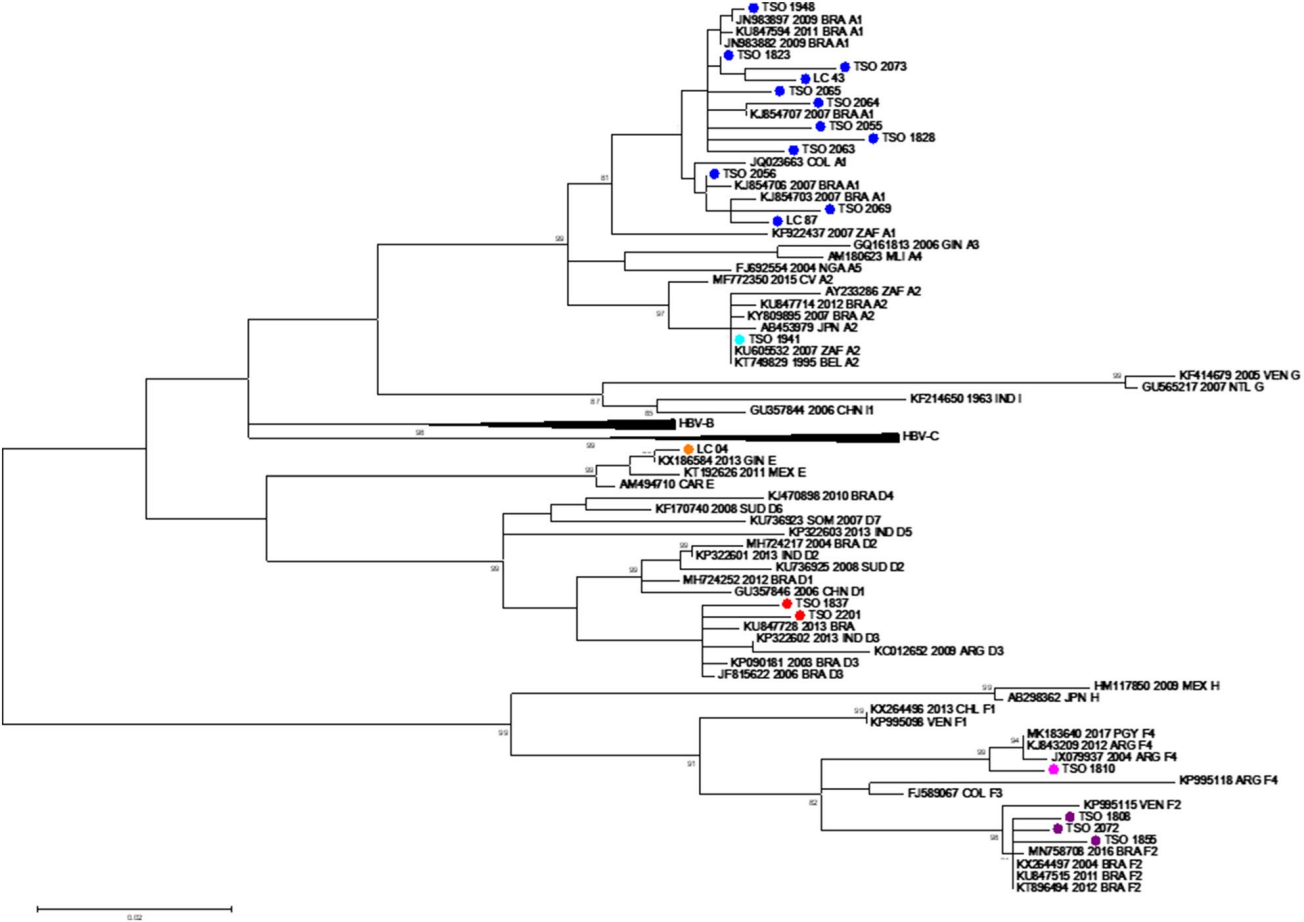
Genotypic distribution of major populations obtained by Sanger sequencing. Maximum likelihood phylogenetic tree composed of 20 serum sequences from this study (marked with colored dots) and 68 reference samples retrieved from GenBank (accession numbers are displayed in the phylogenetic tree).Evolutionary analyses were conducted in MEGA7 and inferred byusing the Maximum Likelihood method based on the GTR + I + G nucleotide substitution model. Genotypes/subgenotypes are represented by the following colors: dark blue: HBV-A1; light blue: HBV-A2; red: HBV-D3; orange: HBV-E; purple: HBV-F2; pink: HBV-F4.

The mean HBV DNA viral load in serum was 5.19 ± 4.3 log10 IU/mL (median 5.29, IQR 3.01–7.93; 4.92). All sera samples (n = 20) were successfully sequenced by both NGS and Sanger methods. However, only seven samples of oral fluid and ten samples of DBS were successfully sequenced by Sanger method. Regarding NGS, seven samples of oral fluid and six samples of DBS had sufficient quality for the quasispecies analyses. The average depth of sequence coverage per nucleotide position from sera samples (n = 20) was 133,793 (minimum 30,434 and maximum 312,225), oral fluid/saliva (n = 7) was 134,082 (minimum 22,561 and maximum 361,230), and DBS (n = 6) was 128,677 (minimum 56,970 and maximum 271,907). The genotype distribution was as follows: A 65% (13/20), D 10% (2/20), E 5% (1/20) and F 20% (4/20). Regarding subgenotypes, among the 13 patients who were infected with genotype A, 12/13 (92.3%) of these strains were classified as subgenotype A1 (HBV/A1), clustering with sequences previously characterized in Brazil from the Asia-American clade^25^, while 1/13 (7.7%) was classified as subgenotype A2 (HBV/A2), clustering with sequences from Brazil, South Africa, and European countries such as Italy and Belgium^26,27^. All genotype D isolates were classified as HBV/D3, grouping with sequences from South Brazil^26,28^. Genotype F was classified as HBV/F2 (3/4; 75%) and HBV/F4 (1/4; 25%), clustering with sequences from Venezuela, Paraguay, and Northern Brazil, respectively^29–31^. HBV/E was found in the serum of an African man and was genetically related to sequences from Guinea (unpublished). Similar topologies were obtained from Sanger (Fig. 1) and NGS phylogenetic trees (not shown).Sanger sequences have been deposited in Genbank (https://www.ncbi.nlm.nih.gov/nucleotide/) under accession numbers OQ190191 to OQ190210.

Genotype agreement between viral sequences in serum and oral fluid paired samples was 100% (genetic distances between paired samples: 0.0–0.006), while agreement between viral sequences in serum and DBS was ~ 80% (genetic distances 0.0–0.115) using both Sanger and NGS. Two individuals (LC-04 and LC-43) displayed discordant genotypes between serum and DBS (Table 2). According to both Sanger and NGS, the LC-04 serum sequence belongs to HBV/E. However, Sanger and NGS major population analyses classified the viral sequence of the LC-04 DBS sample as HBV/A2. The minor population identified by NGS analysis revealed the existence of two distinct haplotypes in the LC-04 DBS sample, thus resulting in mixed populations composed of 91.3% HBV/A2 (major) and 8.7% HBV/E (minor).

**Table 2:** Molecular features of serum, oral fluid, and DBS samples.

Sample	Genotype	Viral load (logUI/mL)	Substitutions	
			Polymerase	HBsAg
1808 serum	F2	2.37	N123D, M129L, S137T, L151F, N246S, T259S, D271E	A159G
1810 serum	F4	3.09	N33S, T118N, H122Y, N123D, Q149K, L151F, D271E	T23I, I25V, L110I, A159G
1823 serum	A1	2.29	N122H, M129L, N131D, V163I, I253V	T131N, S207N, V209L
1828 DBS	A1	NA	I53L, V112I, N122H, **Y126C**, M129L, V142A, V163I, Q215H, I253V	V96A, M103I, T118A, K122R**, T131N, F134L**, **D144A**, V168A, V180A, S207T
1828 oral fluid	A1	NA	I53L, V112I, N122H, **Y126C**, M129L, V142A, V163I, Q215H, I253V	V96A, M103I, T118A, K122R, T131N, **F134L, D144A**, V168A, V180A, S207T
1828 serum	A1	7.8	I53L, V112I, N122H, **Y126C**, M129L, V142A, V163I, Q215H, I253V	V96A, M103I, T118A, K122R, T131N, **F134L**, **D144A**, V168A, V180A, S207T
1837 DBS	D3	NA	L91I, F122L, Q130P, N131H, Y135S, C256S, D263E, I266V, V278I	A159G, S207N
1837 oral fluid	D3	NA	L91I, F122L, Q130P, N131H, Y135S, C256S, D263E, I266V, V278I	A159G, S207N
1837 serum	D3	7.09	L91I, F122L, Q130P, N131H, Y135S, C256S, D263E, I266V, V278I	A159G, S207N
1855 DBS	F2	NA	N123D, M129L, N131D, S137T, L151F, S213T, L220I, N246S, T259S, D271E, T313A	N40S, T45L, C76Y, P127L, A159G, N204K
1855 oral fluid	F2	NA	S74P, N123D, M129L, S137T, S213T, N246S, T259S, D271E, Q288H	T45L, Q101H, P127L, A159G, N204K
1855 serum	F2	6.04	N123D, M129L, N131D, S137T, L151F, S213T, L220I, N246S, T259S, D271E, T313A, I315IM	N40S, T45L, C76Y, P127L, A159G, N204K
1941 oral fluid	A2	NA	L217R, Q319K	T131N
1941 serum	A2	> 9	L217R, Q319K	T131N
1948 oral fluid	A2	NA	N122H, M129L, V163I	T131N, S207N, V209L
1948 serum	A1	7.54	N122H, M129L, V163I, A317S	T131N, S207N, V209L
2055 serum	A1	3.08	I53T, N122H, Y126H, M129L, V163I, I253V, D263E, H271Q	N40S, S45P, T131N, S174N, S207N, V209L
2056 DBS	A1	NA	N122H, M129L, W153R, V163I, I253V, V278I	T131N, S207N, V209L
2056 oral fluid	A1	NA	N122H, M129L, W153R, V163I, I253V, V278I	Q101H, T131N, S207N, V209L
2056 serum	A1	9	N122H, M129L, W153R, V163I, I253V, V278I	T131N, S207N, V209L
2063 serum	A1	4.55	I53S, N122H, M129L, V163I, N238Y, I253V	G44E, S45A, L49R, T131N, S207N, V209L
2064 DBS	A1	NA	R110G, N122H, M129L, V163I, I253V	L175S, T131N, S207N, V209L, P217L
2064 serum	A1	3.79	R110G, N122H, M129L, V163I, I253V	I68T, A194V, T131N, S207N, V209L
2065 DBS	A1	NA	N122H, M129L, W153RW, V163I, M171LM, Q215HQ, I253V, V278I	**Y100C,** T131N, E164EG, A194AV, S207NT, I208IT, V209L
2065 serum	A1	2.72	N122H, M129L, V163I, I253V	**Y100C,** T131N, A194AV, S207T, I208T, V209L
2069 serum	A1	7.3	I53V, N122H, M129L, W153R, S159T, V163I, S219A, I253V, V278I	L21S, L49R, I68T, **Y100C**, T131N, Y161F, S207N, I208T, V209L, S210R
2072 DBS	F2	NA	N123D, M129L, S137T, N246S, T259S, D271E, T313A	T45L, A159G, Y161F
2072 serum	F2	6.66	N123D, M129L, S137T, N246S, T259S, D271E, T313A	T45L, A159G, Y161F
2073 serum	A1	2.53	I53V, S117C, N122H, Q125K, M129L, V163I, I253V	R24K, **L109V**, T131N, V184A, T189I, S204N, S207N, I208T, V209L
2101 oral fluid	D3	NA	N53K, F122L, Q130P, Y135S, D263E, I266V, V278I	T45S, A159G
2101 serum	D3	8.66	N53K, F122L, Q130P, Y135S, D263E, I266V, V278I	T45S, A159G
LC04 DBS	A2	NA	I53INST, T54P, Q67KN, S68T, L217R	S45APST, P46HLPR, S58S, T131N, N59IKRT
LC04 serum	E	3.83	A313S	A159G
LC43 DBS	A1	NA	T53P, N123D, M129L, N134D, S137T, K212R, K241R, N246S, V253I, T259S, D271E	G44A, T45L, S55C, A159G, Y200S, N204D
LC43 serum	F2	2.8	T53P, N123D, M129L, S137T, K212R, K241R, N246S, V253I, T259S, D271E, T313A	T23I, G44A, T45L, S55C, T131N, Y200S, N204D
LC 87 serum	A1	3.26	N122H, M129L, W153R, V163I, I253V, V278I	**Y100C**, **T131N,** A194V, S207N, V209L

*NA* Not available. Clinically relevant substitutions are highlighted in bold.

Regarding LC-43, DBS sequences by Sanger and serum sequences by NGS (major population) classified this isolate as HBV/F2. DBS sequence by NGS was not successfully obtained. Intriguingly, the LC-43 serum sequence by the Sanger method was classified as HBV/A1. Minor population analyses of serum sequence by NGS reveal the existence of distinct haplotypes, however, all of them clustered in the HBV/F2 clade.

### Mutations

Amino acid mutations in polymerase and/or S gene were observed in all 20 HBV serum samples and in their respective oral fluid and/or DBS paired samples, when available. The majority of HBV sequences from serum samples (NGS) demonstrated a high degree of agreement (98.3%) with the sequences obtained by Sanger. However, NGS detected 6% more polymorphisms in the polymerase and HBsAg genes than the Sanger method. Agreement of 100% in the mutation profile was identified between viral sequences in serum and DBS samples by both Sanger and NGS (major population). However, few differences (< 2%) were observed between the sequences of serum/DBS and oral fluid obtained using Sanger and NGS methods. Major population analysis of the reverse transcriptase pointed to positions H122 and M129 as the most polymorphic (≥ 75% variability), followed by V163 (55%) and I253 (50%). There was no statistical correlation between viral loads and genetic diversity, as expressed by the number of polymorphisms per sample. No antiviral primary resistance mutations were found in the polymerase gene in either Sanger sequences or NGS sequences of major population. However, minor population analysis demonstrated the existence of the rtM204I resistance mutation in all individuals at percentages ranging from 2.8 to 7.5% in serum, 2.5–6.3% in oral fluid, and 3.6–7.2% in DBS.

**Figure 2:**
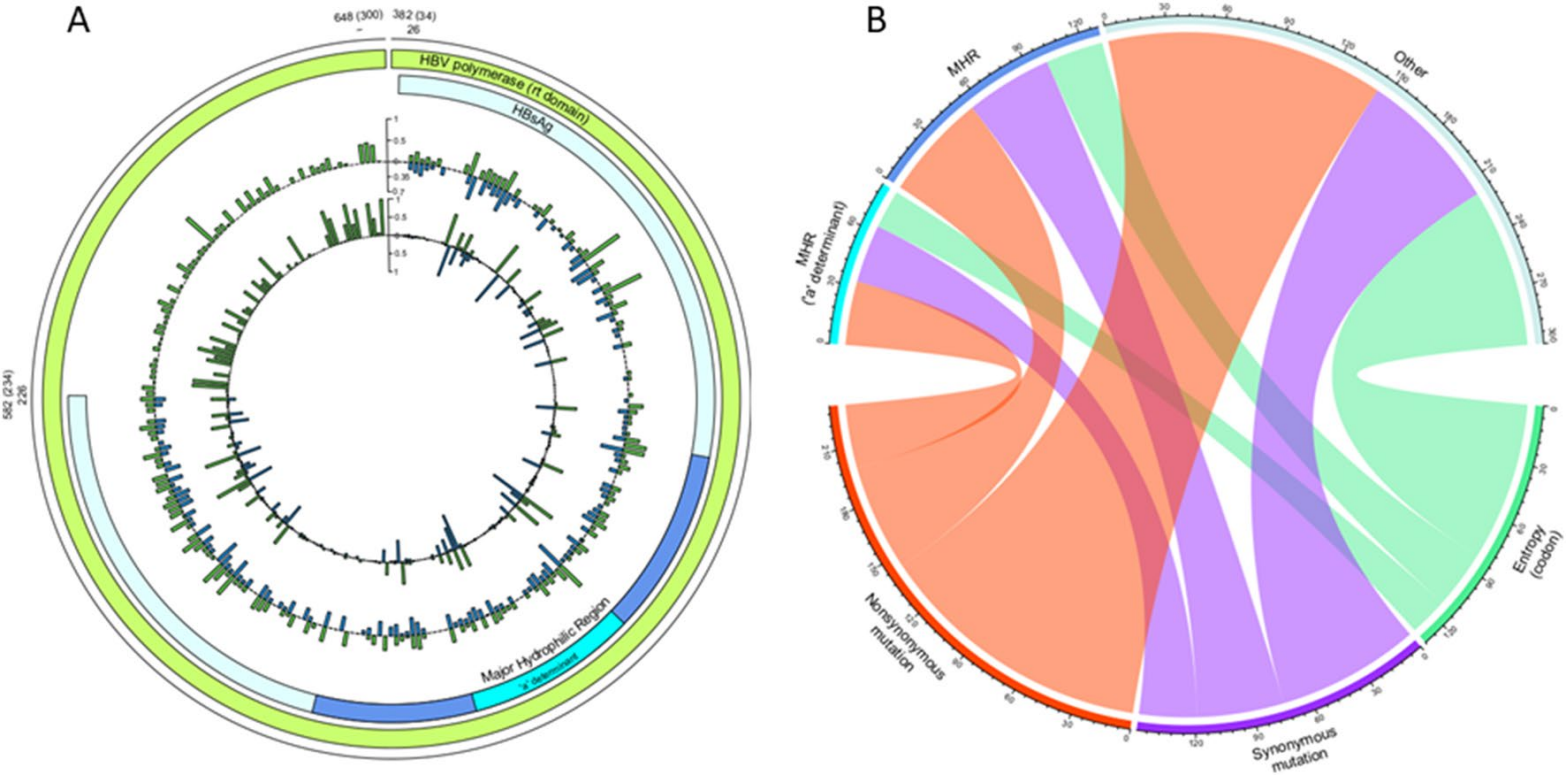
Complexity and diversity features of HBV quasispecies. (**A**) Shannon entropy (outer circle) and mutation frequencies (inner circle) observed in HBV polymerase (RT domain; green) and HBsAg (blue). The frequency of substitutions in both genes is expressed by the scale bar. The (**B**) chord diagram demonstrates the connections between molecular features and HBV functional domains. The proportions of each feature are indicated by the lengths of the Arc.

HBsAg clinically relevant mutations were detected in the viral major population of all individuals, by both Sanger and NGS. Y100C was observed in three subjects. One subject provided only serum sample, in another, this mutation was detected in both serum and DBS samples, while in the third case, Y100C was detected only in oral fluid. The substitution F/P134Y/LD was detected in sequences from all fluids of three subjects; D144A was detected in viral sequences from all fluids of one subject. TheT131N mutation was detected in all but one of the HBV/A sequences. Furthermore, mutations 109 V and Y126C were detected in one individual each (Table 2). Wild-type subpopulations were observed in all individuals in serum, oral fluid, and DBS. Several isolates displayed additional amino acid polymorphisms within the ‘a’ determinant region (aa124-147) of the major hydrophilic region (MHR), with some occurring in lymphocyte B and T binding sites. As shown in Fig. 2A, both complexity and diversity of the subpopulations were similar in polymerase and HBsAg. Hot spots of complexity and diversity (close to 100%) were observed at B and T lymphocyte binding sites in HBsAg (positions 95,111, 123, 142–145, 158, 190, 205). Despite not showing statistical significance, Fig. 2B shows a high enrichment of significant features in ‘other’ regions of HBsAg (excluding MHR and "a" determinant), which had the highest entropy and the highest proportion of non synonymous mutations.

## Discussion

It has been proposed that HBV quasispecies play a role in viral persistence, since genetic diversity serves as a viral reservoir under immune-mediated selection^3,32,33^. In this study, we assessed the molecular features of HBV quasispecies in different biological fluids from chronically infected patients. Our results demonstrated high agreement among HBV major populations from serum, oral fluid and DBS using both Sanger and NGS methods. Although previous studies by our group have demonstrated the accuracy of using alternative fluids such as DBS and oral fluid for HBV molecular analyses^6,7,10^, to the best of our knowledge, this is the first report on the diversity of HBV quasispecies by NGS in alternative fluids.

As observed by previous studies using Sanger sequencing^6,7^, there was a high degree of agreement in the classification of genotype/subgenotype between serum, DBS, and oral fluid (when available). All but two of the paired samples displayed the circulation of the same HBV major population, as also demonstrated by the agreement between Sanger and NGS sequences. However, two individuals presented discordant genotypes between their paired serum and DBS samples, as confirmed by both sequencing methods. Although coinfections by distinct HBV genotypes are relatively common events^34–36^, to the best of our knowledge, the detection of different HBV genotypes in serum and DBS has not yet been documented. Nevertheless, previous studies found discrepancies between HBV populations in different biological compartments^32,34^, suggesting that distinct HBV variants may exhibit tissue tropism specificity. It is known that there is a significant presence of peripheral blood mononuclear cells (PBMCs) in whole blood that are rarely found in serum samples due to centrifugation. Thus, it is possible that DBS samples reflect in part the diversity of HBV present in PBMCs, while serum samples are more likely to mirror the viral populations in hepatocytes. In fact, both major and minor population analyses demonstrated variations in mutation patterns among the three fluids, thus corroborating the hypothesis of viral compartmentalization.

In this study, genotypes A (subgenotypes A1 and A2), D (subgenotype D3), E and F (subgenotypes F2 and F4) were detected. Except for genotype E, which is restricted to Africa, the genotypes found here are endemic in Brazil, as previously reported^36–39^. Most HBV/A1, A2 and D3 sequences clustered with previously described Brazilian sequences^25–28^, while HBV/F2 and F4 grouped with sequences from Latin America such as Venezuela, Paraguay and Brazil^29–31^. No significant differences among viral subpopulations present in serum, oral fluid and DBS (when available) were found, except for two individuals who clearly presented mixed infection. Even though only one haplotype was detected in the serum sampleof patient LC-04 by NGS, minor population analysis demonstrated the cocirculation of two genotypic populations in DBS (91.3% of HBV/A2 and 8.7% of HBV/E). Phylogenetic analysis revealed that the HBV/E viral population found in serum clustered with sequences from Guinea, while the HBV/A2 population found in DBS grouped with sequences from South Africa and Cuba^27,40^. Similar to Brazil, Cuba has a strong migratory relationship with the African continent. Likewise, South Africa is a hub for immigrants from other African countries. From a molecular epidemiological perspective, since patient LC-04 is an immigrant of African origin living in Brazil who is infected with genotypes/variants rarely found in Brazil, it is reasonable to assume that he had acquired the dual infection through allochthonous transmission. On the other hand, in the case of patient LC-43, a Brazilian woman with no history of traveling abroad who presented coinfection with HBV/A1/F2 Brazilian-related genotypes, it is more plausible to assume autochthonous transmission.

It has been demonstrated that during the course of infection, HBV quasispecies are continually subjected to immune and/or drug-mediated selection, experiencing independent evolution processes that may lead to tissue-specific compartmentalization^32,34^. Moreover, as previously suggested, HBV populations that infect PBMCs may be protected to some extent from the selective pressure imposed by antiviral therapy^32^. These observations suggest that extrahepatic sites might represent important sources of HBV escape variants^34,41^.

The genotype discordance found in this study between the subgenotypes detected in serum and in DBS samples was surprising and had, to the best of your knowledge, never been reported before. No evidence of cross-contamination between samples or reagents was detected. All experiments were carried out following strict protocols of good laboratory practices, with appropriate controls. Similarly, no evidence of contamination was observed by comparing these sequences with other samples from this study, although they were extracted and amplified in the same assay. Furthermore, genotype E, which is rarely found in Brazil, was not detected in any other sample.

In this study, most of the amino acid substitutions observed in S/POL genes were not linked to a clinical outcome but rather arose naturally as a result of base misincorporation during reverse transcription. Nonetheless, the most polymorphic sites are present within the MHR region of HBsAg, some of which are in lymphocyte B and T binding sites, demonstrating once again the role of natural selection in the diversity of HBV quasispecies. Polymorphisms in the “a” determinant were detected in major viral populations from 100% of the studied individuals by both Sanger and NGS methods. Clinically relevant mutations such as Y100C, F/P134Y/LD and/or D144A, which have been linked to HBsAg vaccine escape and reduced HBsAg antigenicity, were observed in 20% of the subjects^42–44^. Moreover, all but one HBV/A sample displayed the T131N substitution. Although this substitution is common in HBV/A sequences, previous studies have demonstrated that T131N may reduce HBsAg antigenicity in non-A genotypes by creating an additional N-glycosylation site in the main loop of the “a” determinant^44,45^.

Regarding mutations in RT, although no samples presented primary resistance mutations in major population analysis, all individuals displayed the rtM204I resistance mutation in minor populations, indicating that these individuals might experience therapeutic failure if subjected to strong selective pressure.

This study has some limitations: First, our sample size is limited due to budget constraints for NGS and may not provide definitive information on the potential of oral fluid and DBS to fully reproduce HBV quasispecies diversity in serum. Second, although the analyzed fragment provides necessary information on the presence of clinically relevant mutations, such as vaccine resistance and immune escape, whole genome analysis would be better suited to perform more robust phylogenetic reconstructions and provide a broader overview of HBV quasispecies.

## Conclusions

In conclusion, this study demonstrated that different fluids can be used to assess HBV diversity, nonetheless, genotypic differences according to biological compartments can be observed, denoting the importance of investigating the variability of HBV quasispecies circulating in serum and extrahepatic sites. Further studies involving a large number of alternative samples analyzed by NGS would provide important information about the clinical relevance of HBV quasispecies in different biological fluids.

### Supplementary Information

Supplementary Table S1.

## Data Availability

The datasets supporting the conclusions of this article are included within the article. The datasets generated and/or analysed during the current study are available in the GenBank repository (https://www.ncbi.nlm.nih.gov/nucleotide/), under accession numbers OQ190191 to OQ190210.
