# Supplementary material for: Genetic diversity of hepatitis B virus quasispecies in different biological compartments reveals distinct genotypes
Source: Sci Rep. 2023 Oct 9;13:17023. doi: 10.1038/s41598-023-43655-0 (PMC10562391; doi:10.1038/s41598-023-43655-0)
Supplement: Supplementary file 1 — Supplementary Table S1. [file 41598_2023_43655_MOESM1_ESM.docx]

Table S1: Nucleotide sequences used in phylogenetic analyses.

| Accession number | Genotype | Country |
| --- | --- | --- |
| JN983897 | A1 | Brazil |
| JN983882 | A1 | Brazil |
| JQ023663 | A1 | Colombia |
| KU847594 | A1 | Brazil |
| KJ854703 | A1 | Brazil |
| KJ854706 | A1 | Brazil |
| KJ854707 | A1 | Brazil |
| JN983882 | A1 | Brazil |
| AB453979 | A2 | Japan |
| AY233286 | A2 | South Africa |
| KT749829 | A2 | Belgium |
| KU605532 | A2 | South Africa |
| KU847714 | A2 | Brazil |
| KY809895 | A2 | Brazil |
| MF772350 | A2 | Cape Verde |
| GQ161813 | A3 | Guinea |
| AM180623 | A4 | Mali |
| FJ692554 | A5 | Nigeria |
| AB900112 | B1 | Japan |
| MG372436 | B2 | China |
| KP341009 | B3 | Indonesia |
| KP341010 | B4 | Viet Nam |
| KP659255 | B6 | Canada |
| KP341012 | B7 | Indonesia |
| KP341013 | B9 | Indonesia |
| JX507214 | C1 | Panama |
| JN315779 | C2 | South Korea |
| KU695741 | C3 | Australia |
| KU679960 | C4 | Australia |
| KM999992 | C5 | Philippines |
| EU670263 | C6 | Philippines |
| GU357846 | D1 | China |
| MH724252 | D1 | Brazil |
| MH724217 | D2 | Brazil |
| KP322601 | D2 | India |
| KU736925 | D2 | Sudan |
| JF815622 | D3 | Brazil |
| KP322602 | D3 | India |
| KP090181 | D3 | Brazil |
| KC012652 | D3 | Argentina |
| KU847728 | D3 | Brazil |
| KJ470898 | D4 | Brazil |
| KP322603 | D5 | India |
| KF170740 | D6 | Sudan |
| KU736923 | D7 | Somalia |
| AM494710 | E | Central African Republic |
| KT192626 | E | Mexico |
| KX186584 | E | Guinea |
| KX264496 | F1 | Chile |
| KP995098 | F1 | Venezuela |
| KT896494 | F2 | Brazil |
| KU847515 | F2 | Brazil |
| KX264497 | F2 | Brazil |
| MN758708 | F2 | Brazil |
| KP995115 | F2 | Venezuela |
| FJ589067 | F3 | Colombia |
| JX079937 | F4 | Argentina |
| KJ843209 | F4 | Argentina |
| KP995118 | F4 | Argentina |
| MK183640 | F4 | Paraguay |
| GU565217 | G | Netherland |
| KF414679 | G | Venezuela |
| AB298362 | H | Japan |
| HM117850 | H | Mexico |
| GU357844 | I1 | China |
| KF214650 | I | India |
